# The Intense Cold of January

**Published:** 1852-02

**Authors:** 


// THE WESTERN JOURNAL.

Third Series.—Vol. IX.—No. II.

LOUISVILLE, FEBRUARY 1, 1852.

### THE INTENSE COLD OF JANUARY.

The month just closed was one of the coldest ever experienced in Kentucky. In our next number, we shall be able to present to our readers a complete meteorological table of the month, prepared by Lawrence Young, Esq., of this neighborhood, to whom we were indebted for the table in our last number; and in the mean time we submit a few memoranda of the coldest days.

Monday, the 19th, the mercury, in our Fahrenheit thermometers, stood at 12° below zero, early in the morning, and only rose to within 1° of zero during the day—the mean temperature of the day having been 7° below zero. Tuesday morning, the 20th, we saw it at 13° below zero; but this was not the *minimum* temperature of the morning, for clouds had risen before we made our observation and affected the the termometer. At midnight Prof. Drake had seen it 15° below zero, and some thermometers in the neighborhood indicated a still lower degree. In the neighborhood of Frankfort it was reported to have fallen 20° below zero; and from the following extracts of letters from our correspondents it will be seen that in other parts of the State the cold was still more intense. From Maysville we have the following account:

"On Monday, the mercury stood, at 5 A. M., at 6° below zero, at sunrise at 8°, at 10 A. M. at 5°, at 2 P. M. at 2°, at 8 P. M. at 10°, and at 12 P. M., midnight, at 26° below zero. On Tuesday, at 6 A. M. at 20° below, and at daylight at 16° below zero. On Monday, the range was from 2° to 8° below all day, in *daylight.* The foregoing agrees substantially with several other accounts kept here."

*Flemingsburg*: "Mercury 22° below zero this morning at 6 o'clock, and at John Moore's, in the country, a mile from town, 30° below zero!"

Dr. Fritts, of Carlise writes as follows:

"The weather has been so cold here that I cannot refrain giving you the range of the thermometer for a day or two. January 19, at 6 o'clock, A. M., 6 degrees below zero, and until 6 P. M., the mercury did not rise to zero, remaining all day from 2 to 4 degrees below—the coldest day we ever saw. At 9 P. M., it was 12° below; at 10 it was 14°; and at 2 o'clock on the morning of the 20th, the mercury stood at 20 *degrees below zero!* the coldest, we think, in the memory ot man. I think it quite likely but few persons saw the mercury so low. I was called up at that hour, or I should not have seen it. At 6 o'clock it had moderated very much, being only 10° below!"

But the most intense cold seems to have been felt at Helena, as shown by the following account:

"As the cold of Monday and Tuesday, January 19 and 20, has no parallel, at least for many years in our part of the country, I send you the following table of observations as indicated by my thermometer:

"Monday, daylight, 6 degrees below zero; sunrise, 8 degress below zero; 2 P. M., 2 degrees below zero; 8 P. M,, 15 degrees, and at midnight, 30 degrees below zero.

"Tuesday, 2 A M., 30 degrees below zero; 7 A. M., 16°; 9 A. M., 4°, and M. 0°.

If the accuracy of the instruments used can be relied upon, so intense a degree of cold was probably never before felt in this latitude. The lowest point at which we have ever seen the mercury was 20° below zero, which was on Sunday morning, the 9th of February, 1835.

It may be interesting to some of our readers to compare the late frost with preceding seasons of great severity. We find, in our diary for 1835, the following memoranda of the weather, at Lexington:

'Saturday, February 7th.—At sun-rise, thermometer stands 13° below zero, and the wind is blowing briskly; 8 o'clock, 15° below zero; 9

o'clock, 16° below zero; a strong wind still blows; the air is filled with floating ice, giving the atmosphere a hazy, lurid appearance. It is a dismal morning. People were frost-bitten coming to market. 10 o'clock, the thermometer has risen only 1°, and at 3 o'clock is still 8° below zero, which was the *maximum* for the day. At 5 o'clock, it had fallen 1°, and at 10, P. M., was 14° below zero. The north wind still sweeps along. Such a day was never before seen in Lexington.

'Sunday, February 8th.—At 7 o'clock this morning, the thermometer is 20½° below zero—nearer 21° than 20°. It is perfectly clear; the sun rose bright and red; the wind still blows, but more gently. At 8, 16° below zero; at 9, 15°; at 11, 3°; between 3 and 4 o'clock, it rose to within 1° of zero; but at 6, it was again 5° below.'

The mean temperature of twelve hours, on Saturday, was 11.2° below zero; the mean of the day following was 8° below zero; and the mean of twenty-four hours, from Saturday to Sunday morning, was 13.2° below zero, which, we think, exceeds, a little, the cold ever yet experienced by "the oldest nhabitants" of Kentucky.

During the late frost, wagons and heavily-loaded drays crossed the Ohio river on the ice between Louisville and Jeffersonville, and people were still walking over as late as the 29th.

